# Simultaneous Profiling and Holistic Comparison of the Metabolomes among the Flower Buds of *Panax ginseng*, *Panax quinquefolius*, and *Panax notoginseng* by UHPLC/IM-QTOF-HDMS^E^-Based Metabolomics Analysis

**DOI:** 10.3390/molecules24112188

**Published:** 2019-06-11

**Authors:** Li Jia, Tiantian Zuo, Chunxia Zhang, Weiwei Li, Hongda Wang, Ying Hu, Xiaoyan Wang, Yuexin Qian, Wenzhi Yang, Heshui Yu

**Affiliations:** 1 Tianjin State Key Laboratory of Modern Chinese Medicine, Tianjin University of Traditional Chinese Medicine, Tianjin 300193, China; jia10309@126.com (L.J.); 13553170361@163.com (T.Z.); 18202669277@163.com (C.Z.); lww11413@163.com (W.L.); 17862987156@163.com (H.W.); huying3916@163.com (Y.H.); 14741372177@163.com (X.W.); qyx15232238286@163.com (Y.Q.); 2 Tianjin Key Laboratory of TCM Chemistry and Analysis, Tianjin University of Traditional Chinese Medicine, Tianjin 300193, China; 3 College of Pharmaceutical Engineering of Traditional Chinese Medicine, Tianjin University of Traditional Chinese Medicine, Tianjin 300193, China

**Keywords:** *Panax*, flower bud, UHPLC/IM-QTOF-MS, high-definition MS^E^, metabolomics, chemical marker

## Abstract

The flower buds of three *Panax* species (PGF: flower bud of *P. ginseng*; PQF: flower bud of *P. quinquefolius*; PNF: flower bud of *P. notoginseng*), widely consumed as healthcare products, are easily confused particularly in the extracts or traditional Chinese medicine (TCM) formulae. We are aimed to develop an untargeted metabolomics approach, by ultra-high performance liquid chromatography/ion mobility-quadrupole time-of-flight mass spectrometry (UHPLC/IM-QTOF-MS) to unveil the chemical markers diagnostic for the differentiation of PGF, PQF, and PNF. Key parameters affecting chromatographic separation and MS detection were optimized in sequence. Forty-two batches of flower bud samples were analyzed in negative high-definition MS^E^ (HDMS^E^; enabling three-dimensional separations). Efficient metabolomics data processing was performed by Progenesis QI (Waters, Milford, MA, USA), while pattern-recognition chemometrics was applied for species classification and potential markers discovery. Reference compounds comparison, analysis of both HDMS^E^ and targeted MS/MS data, and retrieval of an in-house ginsenoside library, were simultaneously utilized for the identification of discovered potential markers. Satisfactory conditions for metabolite profiling were achieved on a BEH Shield RP18 column and Vion™ IMS-QTOF instrument (Waters; by setting the capillary voltage of 1.0 kV and the cone of voltage 20 V) within 37 min. A total of 32 components were identified as the potential markers, of which Rb3, Ra1, isomer of m-Rc/m-Rb2/m-Rb3, isomer of Ra1/Ra2, Rb1, and isomer of Ra3, were the most important for differentiating among PGF, PQF, and PNF. Conclusively, UHPLC/IM-QTOF-MS-based metabolomics is a powerful tool for the authentication of TCM at the metabolome level.

## 1. Introduction

The authentication of herbal medicine or traditional Chinese medicine (TCM), that is, to identify the genuine species and differentiate the counterfeits and surrogates, is the most important aspect in quality control [1]. Current approaches for authentication of TCM rely on microscopic features, physicochemical properties, thin-layer chromatography (TLC), high performance liquid chromatography (HPLC), and DNA (deoxyribonucleic acid) sequencing, etc. [2,3]. On the metabolome level, the fingerprint is one of the most powerful tools capable of delineating the holistic profiles. However, conventional approaches, by either TLC or HPLC, are always based on the monitoring of a single or a few markers, which, in some cases, fail to ensure the authenticity of TCM, particularly the TCM formulae that involve multiple constituent drugs. Simultaneous monitoring of multiple markers renders a practical tool in authenticating both the raw TCM materials and TCM formulae [4]. It has been demonstrated that selective ion monitoring (SIM) of multiple chemical markers can achieve the qualitative identification of all constituent drugs in a formula, or identification of a single TCM species simultaneously from different formulae [5,6]. However, in this context, the premise is how to discover the potential marker compounds from the complex matrix with both the abundant and minor components being taken into account. Holistic evaluation strategies thus are in great need.

Untargeted metabolomics has become a mature approach in holistically exploring the differential components from different chemical groups at the metabolome level, and covers extensive applications in the authentication of TCM. The troublesome issues, such as differentiation among the easily confusing species [7,8], different geographic origins [9], different parts from the same plant [10], different ages [11,12], and different processing technologies [13], can be accomplished by untargeted metabolomics workflows. Untargeted metabolomics is typically performed following four steps: (i) acquisition of metabolic features by direct infusion or chromatography/MS hyphenation techniques; (ii) deconvolution including peak alignment and peak picking generating a peak table; (iii) multivariate statistical analysis by unsupervised or supervised classifiers to unveil the components associated with groups segregation, and (iv) quantitative assay or validation process to rationalize the discovered chemical or biomarkers. In addition to the most preferable reversed-phase HPLC or UHPLC (ultra-high performance liquid chromatography), HILIC (hydrophilic interaction chromatography) and SFC (supercritical fluid chromatography) can offer more metabolic feature information beneficial to the comprehensive assessment [14,15]. Considering the occurrence of ion suppression in untargeted MS mode, quantitative metabolomics can overcome the deficiency and more truly reflect the content difference of metabolites by multiple reaction monitoring (MRM) or parallel reaction monitoring (PRM) [16]. Pseudo-targeted metabolomics, by integrating the merits of untargeted profiling and MRM detection, becomes a potent omics tool having great application potential [17,18].

Aiming to enable comprehensive profiling and characterization of the multicomponents from TCM, new advances have emerged providing enhanced dimensions in both chromatography and MS. On one hand, multi-dimensional liquid chromatography (MDLC) operating in either on-line or off-line mode can greatly improve the peak capacity and selectivity, allowing the resolution and detection of more minor components [19,20]. On the other hand, enhanced MS^n^ strategies, such as precursor ions list-triggered data-dependent acquisition [21,22] or non-biased data-independent acquisition (such as MS^E^, AIF, SWATH, etc.) [10,14,23,24], can remarkably improve the coverage in characterizing interested metabolites. High-resolution MS with the ion mobility function (e.g., SYNAPT series and Vion IMS-QTOF from Waters, 6560 QTOF from Agilent) provides an additional dimension in separating ions based on the size, shape, and charge, aside from the generation of high-accuracy MS data [25,26]. Therefore, the platform, UHPLC/IMS-QTOF-MS, can offer four-dimensional structure information (t_R_, drift time, MS, and MS^2^) supporting the systematic multicomponent characterization of TCM.

Multiple species from the *Panax* genus have exhibited tonifying effects to human health, of which *P. ginseng* C.A. Meyer (Asian ginseng), *P. quinquefolius* L. (American ginseng), *P. notoginseng* (Burk.) F.H. Chen (Sanchi ginseng), *P. japonicus* C.A. Meyer (Japanese ginseng), and *P. vietnamensis* Ha & Grushv. (Vietnamese ginseng), are the most reputable and most popular worldwide [27]. Phytochemical researches have isolated versatile primary and secondary metabolites from this genus, such as the saponins, polysaccharides, flavonoids, amino acids, organic acids, and sterols, etc. The saponins (ginsenosides) thereof are a class of rich, specific, and biologically active substances closely associated with the therapeutic effects on the cardiovascular and immune systems [28,29]. The saponins isolated from the *Panax* genus have unique structure features: (1) bearing a tetracyclic dammarane (or its derivatives) or pentacyclic oleanolic acid sapogenin; (2) glycosylated with glucose (Glc), glucuronic acid (GluA), rhamnose (Rha), xylose (Xyl), and arabinose (Ara) in either a furan or pyran form; and (3) substituted with acyl groups (e.g., acetyl, malonyl, butenyl, and octenyl, etc.) [27]. According to the TCM theory, Asian ginseng, American ginseng, and Sanchi ginseng, have differentiated natural properties and clinical applications [4]. Aiming to unveil the underlying mechanism, collections of chemical analysis works have been conducted to compare the differences in the compositions of saponins [4], fatty acids [30], and lipidomes [15], present in *P. ginseng*, *P. quinquefolius*, *P. notoginseng*, and some other congeneric species. It is noted that, in addition to the root (and rhizome), the stem leaf, flower bud, and berry of *P. ginseng*, *P. quinquefolius*, and *P. notoginseng*, have saponins in abundance as well [10,31,32,33]. The flower buds of *P. ginseng* (PGF), *P. quinquefolius* (PQF), and *P. notoginseng* (PNF), despite having not been recorded in Chinese Pharmacopoeia (2015 edition), are extensively consumed as healthcare products and are increasingly attracting attention due to their potential medicinal use [34]. Although PGF, PQF, and PNF, have individual appearance features, it becomes very difficult to differentiate them in the extracts and the materials used in healthcare products and TCM formulae. By a comprehensive retrieval of the literature, despite a report has quantitatively compared the contents of some major PPD- and PPT-type ginsenosides together with their malonyl forms [35], unfortunately, a systematic comparison among PGF, PQF, and PNF in respect of the whole metabolome, has never been documented.

**Figure 1:**
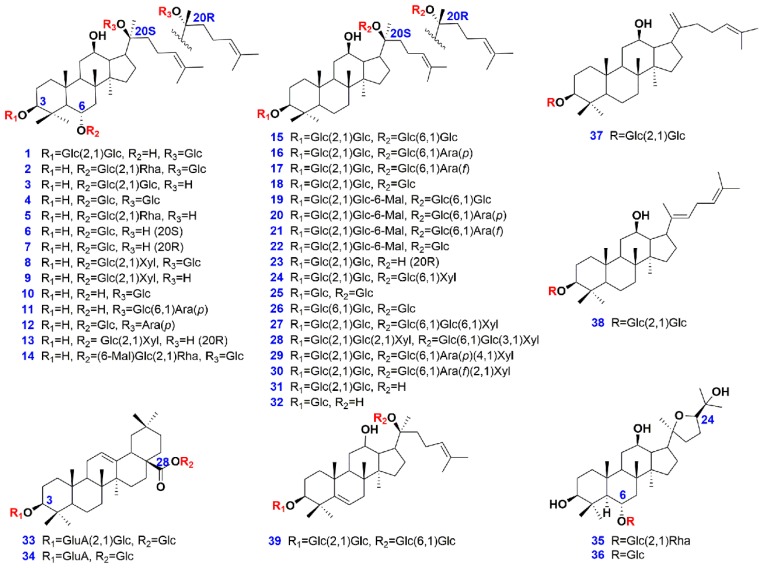
Chemical structures of 39 ginsenoside reference compounds.

The aim of this work was to develop an untargeted metabolomics approach, by UHPLC/IM-QTOF-MS (ion mobility-quadrupole time-of-flight mass spectrometry), to systematically probe into the metabolome difference among PGF, PQF, and PNF. The conditions for chromatographic separation and MS detection on Vion IMS-QTOF were optimized to enable well the resolution and sensitive monitoring of the multicomponents. Forty-two batches of the flower bud samples were analyzed under the optimal UHPLC/IM-QTOF-MS condition. Progenesis QI was utilized for streamlined component extraction (peak alignment and peak picking), while pattern recognition approaches PCA (principle component analysis) and OPLS-DA (orthogonal partial least squares discriminant analysis) were applied to discover potential markers which were further identified by multiple approaches, such as comparison with the reference compounds (Figure 1 and Table S1), analyzing the HDMS^E^ and targeted MS/MS data, and searching against an in-house ginsenoside library (consisting of 504 compounds). Hopefully, the results obtained would be useful to exactly differentiate the flower buds of three *Panax* species, and thus better benefit the quality control of ginseng both as the raw drug materials and their use in TCM formulae.

## 2. Results and Discussion

### 2.1. Optimization and Development of a UHPLC/IM-QTOF-HDMS^E^ Approach

Aiming to explore the metabolome difference among three flower bud samples (PGF, PQF, and PNF), the first step was to establish a UHPLC/IM-QTOF-MS approach to enable comprehensive metabolites profiling. For this purpose, chromatographic conditions using reversed-phase (RP) columns (involving stationary phase, mobile phase, column temperature, and gradient elution program) and MS parameters using a high-resolution hybrid Vion™ IMS-TOF mass spectrometer (capillary voltage, cone voltage, and collision energy) were optimized using a QC sample.

**Figure 2:**
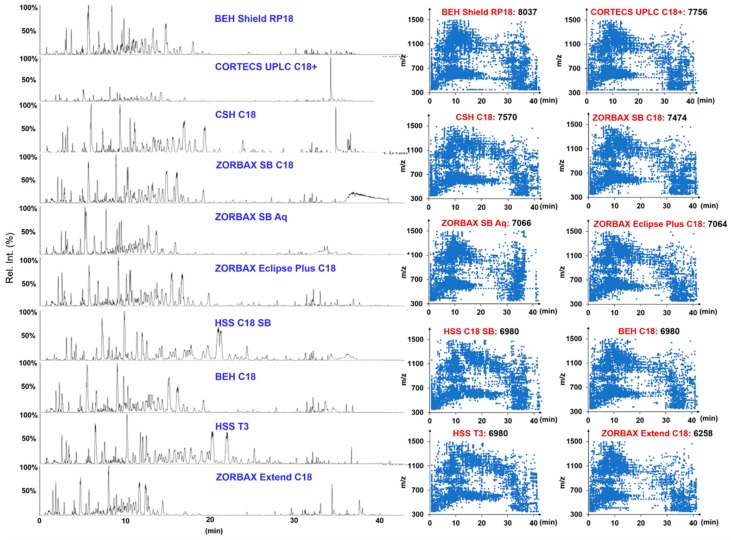
Selection of the stationary phase for the ultra-high performance liquid chromatography (UHPLC) separation of metabolites from the flower bud of *P. ginseng*, (PGF), the flower bud of *P. quinquefolius* (PQF), and the flower bud of *P. notoginseng* (PNF). The left shows the base peak chromatograms (BPC) obtained on ten candidate sub-2 µm particles packed columns and the right is the scatter plot of the resolved peaks.

Our previous studies revealed that different additives in the mobile phase (ammonium acetate, AA; formic acid, FA) could largely influence the selectivity of ginsenosides on RP columns [4,10,20,32]. Here, we first compared the influence of AA and FA as the additive on the resolution of ginsenosides using an HSS T3 column (2.1 × 100 mm, 1.8 µm) under each optimized gradient program using QC1 (prepared by mixing the same volume of the test solutions of PGF-2, PQF-14, and PNF-14, Table S2). As witnessed in Figure S1, by injecting the same volume, many more peaks could be resolved when 0.1% FA was added in the mobile phase, but the ion response was not remarkably reduced. Accordingly, ACN/0.1% FA-H_2_O was selected as the mobile phase for further optimization. We subsequently evaluated the effects of different RP columns on the separation of ginsenosides by comparing the number of resolvable peaks and the peak symmetry. Ten UHPLC columns packed with sub-2 µm particles from two vendors (Waters and Agilent), including HSS T3, HSS C18 SB, CSH C18, BEH Shield RP18, BEH C18, CORTECS UPLC C18+ (Waters, Milford, MA, USA), ZORBAX Extend C18, ZORBAX SB-C18, ZORBAX Eclipse Plus C18, and ZORBAX SB-Aq (Agilent Technologies, Waldbronn, Germany), were examined under 35 °C. These columns differ in the silica gel core (fully porous or core-shell), bonding technologies, and bonding groups. As shown in Figure 2, using a unified gradient elution program, the BEH Shield RP18 (8037), CORTECS UPLC C18+ (7756), and CSH C18 (7570) columns, could separate more peaks than the others. Despite the whole peak distribution through the entire spectrum facilitated by BEH Shield RP18 was not as satisfactory as that obtained by HSS T3, it could still resolve the most peaks, and simultaneously had a large potential for further improvement by optimizing the gradient. More importantly, a pair of critical marker ginsenosides for discriminating *Panax* species, Rg1/Re, showed weak separation around 5 min. We thereby selected the BEH Shield RP18 column in this experiment. Notably, in our previous work, BEH Shield RP18 was also the best choice for separating quinochalcones and flavonoids from Carthamus tinctorius [36]. The effects of column temperature varying from 25 °C to 40 °C, were further assessed (Figure S2). Surprisingly, alternation of the temperature only exerted litter influence on the separation of major ginsenosides. A very small difference could be observed in the region of the spectra: <5 min (polar saponins and some flavonoid glycosides) and 20–30 min (PPD and malonylginsenosides). Judged by the resolved peaks, the temperature at 35 °C (8451) was better than those obtained under the other settings (8251 at 25 °C, 8246 at 30 °C, and 8146 at 40 °C). Accordingly, the column temperature was set at 35 °C. By further fine adjustment of the elution gradient, the best chromatography was obtained, with the resolved peaks increasing to 8750.

**Figure 3:**
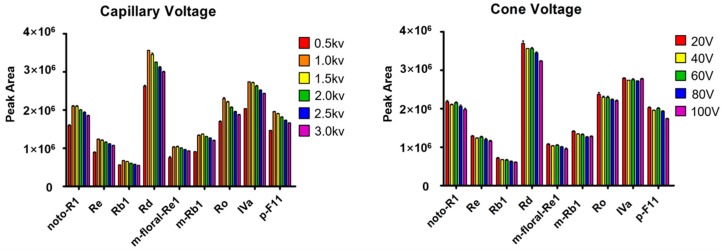
Optimization of two key ESI source parameters of the Vion IMS-QTOF high-resolution mass spectrometer by nine ginsenosides representative of five subclasses. Protopanaxatriol type (PPT): notoginsenoside R1 (noto-R1) and ginsenoside Re (Re); protopanaxadiol type (PPD): ginsenosides Rb1 (Rb1) and -Rd (Rd); oleanolic acid type (OA): ginsenoside Ro (Ro) and chikusetsusaponin IVa (IVa); octillol type (OT): 24(*R*)-pseudoginsenoside F11 (p-F11); malonylated: malonylfloralginsenoside Re1 (m-floral-Re1) and malonylginsenoside Rb1 (m-Rb1). Capillary voltage: 0.5–3.0 kV; cone voltage: 20–100 V.

Key ion source parameters were optimized to enhance the sensitivity in detecting ginsenosides by Vion IMS-QTOF. Two parameters affecting the ion response, including capillary voltage and cone voltage, were optimized by single-factor experiments to improve the sensitivity in detecting ginsenosides. Nine ginsenosides representative of five subclasses of Ginseng saponins, involving noto-R1 and Re (PPT), Rb1 and Rd (PPD), Ro and chikusetsusaponin IVa (OA), 24(R)-p-F11 (OT), m-floral-Re1 and m-Rb1 (malonylated), were used as the index by comparing the peak areas in extracted ion chromatogram (EIC). Six different levels of capillary voltage, including 0.5 kV, 1.0 kV, 1.5 kV, 2.0 kV, 2.5 kV, and 3.0 kV, were compared using the data in triplicate. Results showed all the neutral ginsenosides, including noto-R1, Re, Rb1, Rd, and 24(R)-p-F11, gave FA-adducts as the base peak ions in spite of their different sapogenins, while acidic saponins involving two malonylated (m-florel-Re1 and m-Rb1) and two OA type-ginsenosides (Ro and chikusetsusaponin IVa) generated the highest intensities of deprotonated molecules. The most intense precursor ions were extracted and their peak area values were used for evaluation. We found the variations in peak areas through three parallel injections for all nine ginsenosides were very minor (RSD < 5.0%), which indicated stable performance for the established UHPLC/IM-QTOF-MS approach. As exhibited in Figure 3, seven compounds (noto-R1, Re, Rb1, Rd, Ro, chikusetsusaponin IVa, and 24(R)-p-F11) belonging to four ginsenoside subclasses displayed the highest response when capillary voltage was set at 1.0 kV, and in contrast, two malonylginsenosides (m-floral-Re1 and m-Rb1) were best ionized at 1.5 kV. The intensity differences of the deprotonated precursors for these two malonylginsenosides, determined under capillary voltage 1.0 kV and 1.5 kV, were actually very little. Therefore, the capillary voltage of 1.0 kV was adopted. Cone voltage is an ion source parameter ensuring efficient ion transmission, which can also cause in-source CID disadvantageous for the purpose of structural elucidation [10,37]. In the current work, the cone voltage varying from 20–100 V was evaluated on the intensity of nine ginsenosides (Figure 3). The most intense response of all nine ginsenosides was exclusively observed at the cone voltage of 20 V. While it increased to 100 V, severe in-source fragmentation was observed for the malonylginsenosides. We finally set cone voltage at 20 V. As a result, more sensitive monitoring of ginsenosides from PGF, PQF, and PNF, was achievable by setting capillary voltage and cone voltage at 1.0 kV and 20 V, respectively. By the optimization, the ion response for nine ginsenosides was enhanced by 6.5–17.7%. Ramp collision energy, rather than a single value, can better fragment the gas-phase ginsenoside molecules conjugated with different numbers of sugar groups [10,37]. Ramp energy ranges, including 20–40 eV, 40–60 eV, 60–80 eV, 80–100 eV, 100–120 eV, and 120–140 eV, were examined in the first step using the QC1 sample by observing the richness of product ions for ginsenosides Rb1 and Re, particularly the sapognin-related ion species (Figure S3). We could deduce that, higher levels of collision energy ramps benefitted the elimination of sugars from the saponin molecules, and remarkably differentiated richness in product ions was witnessed for the saponin involving different numbers of sugars. The collision energy ramp, 80–100 eV, could well fragment monoglycosidic, diglycosic, and triglycosic saponins, while a voltage higher than 100–120 eV was necessary for the dissociation of tetra-glycosides. To enable sufficient fragmentation of all saponins involving one to six sugars, we determined to set a wide ramp collision energy at 80–120 eV, under which the product ions were rich for identifying their structures. Because of the availability of ion mobility separation on the Vion IMS-QTOF instrument, we further compared the performance of MS^E^ and HDMS^E^ (IMS function enabled) in the negative mode using the QC1 sample (Figure S4). The differences of two data acquisition modes were embodied in two aspects: i) the ion intensity; and ii) the quality of MS^2^ data. Using nine ginsenoside compounds as the index, the peak area of each component obtained in MS^E^ was 1.65 (Ro) to 18.31 (m-floral-Re1)-folds as those obtained in HDMS^E^. On the other hand, the primary identification results (using UNIFI by searching against the in-house ginsenoside library) from the MS^E^ and HDMS^E^ data were 629 and 779, respectively. We could ascribe these differences to the fact that the co-eluting ions got further separation by IMS, leading to the detection of more peaks but in lower abundance [38].

Ultimately, an optimal UHPLC/IM-QTOF-HDMS^E^ condition for analyzing the ginsenosides from PGF, PQF, and PNF, was obtained as follows: BEH Shield RP18 column kept at 35 °C using 0.1% FA in acetonitrile/0.1% FA in water as the mobile phase; negative HDMS^E^ mode by setting capillary voltage at 1.0 kV, cone voltage at 20 V, and ramp collision energy at 80–120 eV.

### 2.2. Systematic Comparison of the Metabolome Difference among PGF, PQF, and PNF by Untargeted Metabolomics

**Figure 4:**
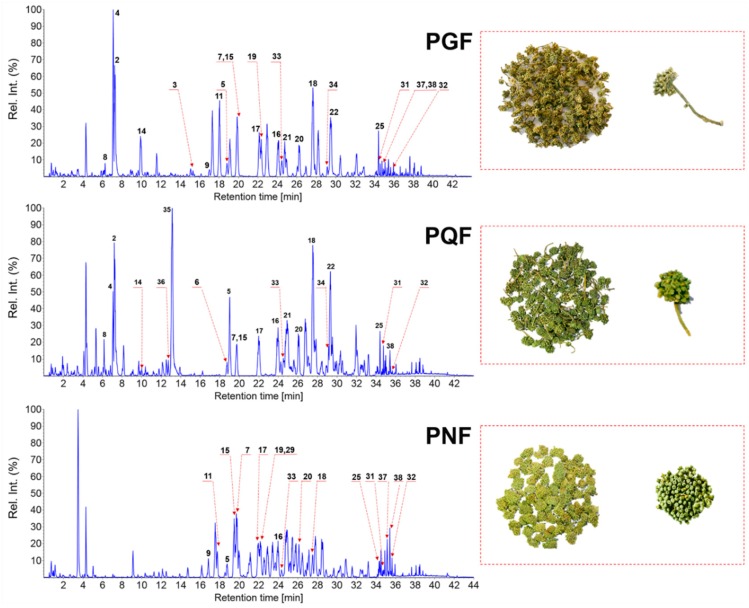
Optical photographs and base peak chromatograms of representative samples in the negative high-definition MS^E^ (HDMS^E^) mode. PGF: flower bud of *P. ginseng*, PGF-12), PQF: flower bud of *P. quinquefolius*, PQF-8), and PNF: flower bud of *P. notoginseng*, PNF-9) Peaks identified by comparing with the reference compounds are annotated in base peak chromatograms with the numbers consistent with Figure 1.

Untargeted metabolomics has been a powerful tool in quality evaluation of TCM enabling the holistic assessment of the whole metabolome [1]. First, metabolite profiling of 42 batches of PGF/PQF/PNF samples was performed by UHPLC/IM-QTOF-HDMS^E^ under optimized conditions. Second, the obtained multi-batch HDMS^E^ data were processed by the Progenisis QI software (Waters Corporation) to perform component extraction, which finally generated a data matrix containing the information of t_R_, m/z, CCS, and normalized abundance [10,14]. Third, the data matrix suffered from further process by “80% rule” and “30% variation” [39,40]. Fourth, the resulting data matrix was input into the SIMCA-P software for pattern recognition chemometrics analysis by PCA (unsupervised) and OPLS-DA (supervised). Variable importance in projection (VIP) ranking could reveal the potential marker compounds by setting a rational cutoff value. The photos of representative flower bud samples and the corresponding base peak chromatograms are shown in Figure 4. The appearance of PGF, PQF, and PNF, showed several differentiated features in respect of inflorescence morphology, length and number of pedicel, etc. [41]. In detail, PNF has a relatively big, mushroom-like, and compact inflorescence with relatively short and bushy flowers, which is different from both PGF and PQF. Pedicel length of PGF and PQF is different, and the former is longer.

**Figure 5:**
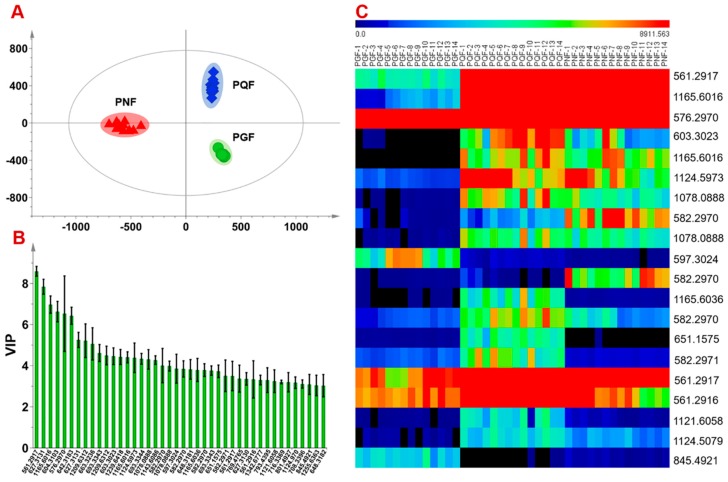
Comparison of the flower buds of *Panax ginseng* (PGF), *P. quinquefolius* (PQF), and *P. notoginseng* (PNF) by chemometrics. (**A**): score plot of OPLS-DA; (**B**) variable importance in projection (VIP) plot of 42 ions with a cutoff set at 3.0; (**C**) heat map plotted by species VS 20 differential ions.

The multi-batch HDMS^E^ data processed by Progenesis QI generated a list consisting of 13,270 ions. Further screening by “80% rule” and “30% variation”, 1506 ions were retained, the normalized abundance of which was used as the variables for further chemometrics analysis using PCA and OPLS-DA. Unsupervised PCA can directly reflect the differentiations among different chemical groups and find outliers [10]. The score plot of PCA (Figure S5) could demonstrate the acceptable stability of the established approach based on the clustering of QC data. On the other hand, the clusters representative of PGF, PQF, and PNF, got well separated, which could indicate relatively large chemical difference, as witnessed in Figure 4. Subsequently, supervised OPLS-DA and VIP (variable importance in projection) plot were utilized to discover the differential components among three different flower bud samples. The established OPLS-DA model displayed good fitness (R^2^X 0.826, R^2^Y 0.983) and predictivity (Q^2^ 0.98), in which each group was remarkably separated with each other (Figure 5A). A total of 42 ions were screened when a VIP cutoff was set at 3.0 (Figure 5B), of which 22 ions were considered as the potential characteristic components (because they are commonly present in at least one species, but not detectable in the other species) and the other 20 (differential ions) were detected in all three species but in differentiated abundance. Variations of 20 differential ions among the tested 42 batches of samples were intuitively reflected in a heat map (Figure 5C).

### 2.3. Identification of the Potential Marker Compounds

The discovered differential ions were characterized to find potential metabolite markers. Multiple solutions, including reference compounds comparison (t_R_, MS, MS^2^, and IM-derived CCS), comparative analysis of the high-accuracy HDMS^E^ and targeted MS/MS data, and the retrieval of an in-house ginsenoside library, were utilized to identify the components consistent with 42 ions. Notably, the CCS values derived from ion mobility separation can provide an additional dimensional evidence to enable more reliable characterization of metabolites [15,38,42]. As a result, 42 ions showing VIP > 3.0 were ascribed to 32 potential marker compounds (Table S3), with their identities and content differences given in Table 1.

**Figure 6:**
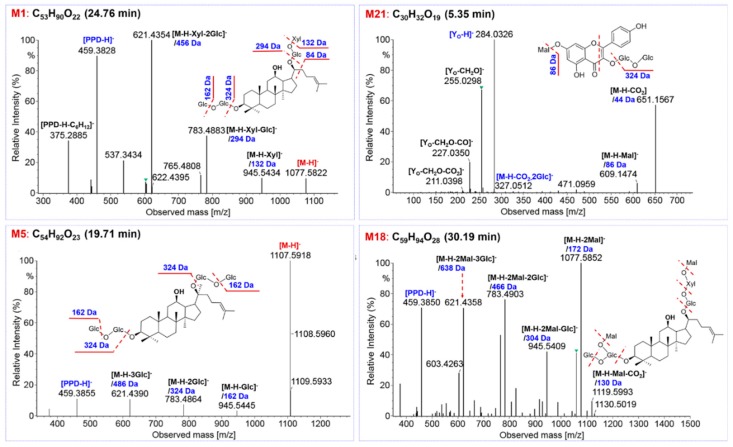
Structural elucidation of four marker compounds based on the negative CID-MS^2^ data. **M1** (Rb3) and **M5** (Rb1) was identified with the aid of reference compounds.

These 32 potential marker compounds, based on the differentiated sapogenins, could be classified into PPD- (**M1**, **M2**, **M4–8**, **M10**, **M13**, **M20, M23**, **M25**, **M26**, and **M30**), PPT- (**M24** and **M31**), malonylated (**M3**, **M9**, **M11**, **M12**, **M14–19**, **M22**, and **M32**), OA- (**M27**), OT- (**M29**), and other types (**M21** and **M28**). Evidently, PPD-type neutral ginsenosides (14 in total) and malonyl PPD-type ginsenosides (12) were the most important markers. Five PPD-type ginsenosides, **M1** (Rb3, t_R_ 24.76 min, C_53_H_90_O_22_; Figure 6), **M2** (Ra1, t_R_ 22.09 min, C_58_H_98_O_26_), **M5** (Rb1, t_R_ 19.71 min, C_54_H_92_O_23_; Figure 6), **M23** (Rc, t_R_ 21.91 min, C_53_H_90_O_22_), **M26** (Rb2, t_R_ 23.90 min, C_53_H_90_O_22_), were identified with the aid of reference compounds. By analyzing the MS^2^ data, we could include that, the characteristic neutral loss (NL 162.05 Da for Glc, 146.06 Da for Rha, 132.04 Da for Xyl and Ara) and characteristic sapogenin ions (m/z 459.38 and 375.29) were diagnostic for the characterization of the other nine unknown marker compounds belonging to this type (**M4**, **M6–8**, **M10**, **M13**, **M20**, **M25**, and **M30**) [20,43]. The flower buds of some *Panax* species contain abundant malonylginsenosides, which are featured by the acylation with one or two polar malonyl groups and have shown potential anti-diabetes effect [10,27,32,43,44,45]. Ten malonylginsenosides (**M3**, **M9**, **M12**, **M14–17**, **M19**, **M22**, and **M32**) and two dimalonylginsenosides (**M11** and **M18**) were identified as the markers. Characteristic fragmentation pathways for malonylginsenosides were neutral elimination of CO_2_ (43.99 Da) and the whole malonyl group (86.00 Da), while dimalonylginsenosides were featured by neutral losses of Mal. (86.00 Da), Mal. + CO_2_ (129.99 Da), 2 × Mal. (172.00 Da), and 2 × Mal + H_2_O. (190.01 Da) (see **M18** in Figure 6). In addition, **M24** (F3, t_R_ 17.89 min, C_41_H_70_O_13_)/**M31** (Rf, t_R_ 15.00 min, C_42_H_72_O_14_), **M27** (chikusetsusaponin IVa, t_R_ 29.13 min, C_42_H_66_O_14_), and **M29** (24R-pseudoginsenoside F11, t_R_ 13.10 min, C_42_H_72_O_14_), were marker ginsenosides belonging to the PPT-, OA-, and OT-type, respectively, which all got identified by comparing with the reference compounds. **M21** (t_R_ 5.35 min, C_30_H_32_O_19_; m/z 651.1567 [M-H-CO_2_]^−^) was identified as a malonyl flavonoid O-glycoside. Characteristic MS/MS features on the malonyl moiety were observed in its MS^2^ spectrum, including m/z 651.1567 ([M-H-CO_2_]^−^) and 609.1474 ([M-H-Mal.]^−^). The remaining MS^2^ fragments at m/z 429.0839 ([M-H-Mal.-Glc-H_2_O]^−^), 327.0512 ([M-H-CO_2_-2Glc]^−^), 285.0404 ([Y_0_]^−^)/284.0326 ([Y_0_-H]^−^), and 255.0298 ([Y_0_-CH_2_O]^−^), 227.0350 ([Y_0_-CH_2_O-CO]^−^), 211.0398 ([Y_0_-CH_2_O-CO_2_]^−^), and 151.0038 (^0.3^A^−^ of RDA), could inform the presence of two Glc and more possibly a kaempferol skeleton (Figure 6). Unfortunately, **M28** (t_R_ 19.55 min), showing a precursor ion at m/z 716.3369, could not be characterized, since no hit was observed in the in-house ginsenoside library and the MS^2^ data were difficult to interpret. Further confirmation of these unknown markers will be performed in future work by the aid of phytochemical isolation and NMR analyses.

**Figure 7:**
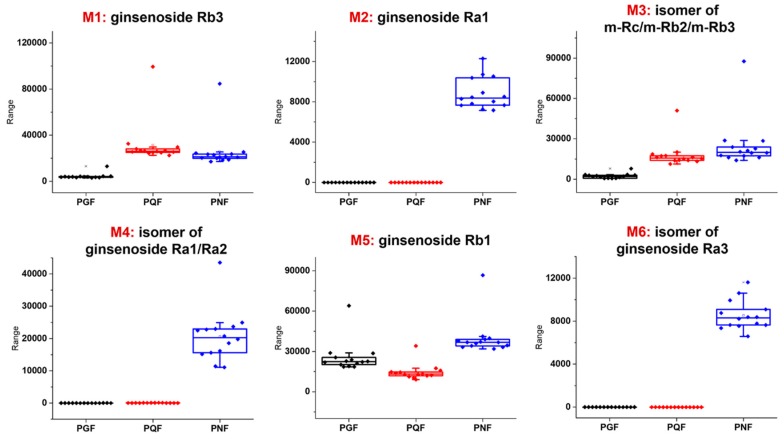
Box charts illustrating the distribution difference of the six most important marker compounds among PGF, PQF, and PNF. **M2**, **M4**, and **M6** can be the characteristic components of PNF.

The content differences of the identified markers among three flower bud samples (PGF, PQF, and PNF) were analyzed. Amongst these markers we could identify that 17 compounds were considered as the characteristic components (corresponding to 22 ions showing VIP > 3.0), while the other 15 components mainly differed in abundance. According to VIP ranking, Rb3, Ra1, an isomer of m-Rc/m-Rb2/m-Rb3, an isomer of Ra1/Ra2, Rb1, and an isomer of Ra3, were the most important markers. Their distribution difference among PGF, PQF, and PNF, is reflected in Figure 7. For these six markers, Ra1 (**M2**), isomer of Ra1/Ra2 (**M4**), and isomer of Ra3 (**M6**), could be three characteristic components for PNF. These characteristics are very important in rapid differentiation among the flower buds of three *Panax* species. We would further confirm these markers by absolute quantitative assays in future. Currently no documents are available that report the differences of PGF, PQF, and PNF, in pharmacology and clinic use. Our findings in this work would benefit understanding of their tonifying effects and, to some extent, advance researches on their medicinal use.

## 3. Materials and Methods

### 3.1. Reagents and Chemicals

Thirty-nine compounds (Figure 1 and Table S1), involving vinaginsenoside R4 (**1**), ginsenosides Re (**2**), -Rf (**3**), -Rg1 (**4**), -Rg2 (**5**), -Rh1 (**6**), 20(*R*)-Rh1 (**7**), notoginsenosides R1 (**8**), -R2 (**9**), ginsenosides F1 (**10**), -F3 (**11**), 20(*S*)-sanchirhinoside A3 (**12**), 20(*R*)-notoginsenoside R2 (**13**), malonylfloralginsenoside Re1 (**14**), ginsenosides Rb1 (**15**), -Rb2 (**16**), -Rc (**17**), -Rd (**18**), malonylginsenosides Rb1 (**19**), -Rb2 (**20**), -Rc (**21**),-Rd (**22**), 20(*R*)-ginsenoside Rg3 (**23**), ginsenosides Rb3 (**24**), -F2 (**25**), notoginsenosides K (**26**), -R4 (**27**), -T (**28**), ginsenosides Ra1 (**29**), -Ra2 (**30**), 20(*S*)-ginsenoside Rg3 (**31**), 20(*S*)-ginsenoside Rh2 (**32**), ginsenoside Ro (**33**), chikusetsusaponin IVa (**34**), 20(*R*)-pseudoginsenosides F11 (**35**), -Rt5 (**36**), ginsenosides RK1 (**37**), -Rg5 (**38**), and 5,6-didehydroginsenoside Rb1 (**39**), purchased from Shanghai Standard Biotech. Co., Ltd. (Shanghai, China) or isolated from the root of *P. ginseng*, were used as the reference compounds in this study. HPLC-grade acetonitrile, methanol (Fisher, Fair Lawn, NJ, USA), formic acid (Sigma-Aldrich, MO, Switzerland), and ultra-pure water in-house prepared using a Milli-Q water purification system (Millipore, Bedford, MA, USA), were used. Detailed information with respect to 42 batches of samples, analyzed in this work, is provided in Table S2. Specimens were deposited at the authors’ lab in Tianjin University of Traditional Chinese Medicine (Tianjin, China).

### 3.2. Sample Preparation

To each of the flower bud sample of three *Panax* species, 50 mg of the accurately weighed powder was soaked in 10 mL 70% aqueous methanol (*v*/*v*). Samples were extracted on a water bath at 25 °C with ultrasound assistance for 1 h. The lost weight was compensated with 70% methanol. After 10-min centrifugation at 14,000 rpm, the supernatant was used as the test solution of 42 batches of samples with the final concentration at 5 mg/mL. A QC sample (QC1) by mixing the equal volume of three representative samples (PGF-2, PQF-14, and PNF-14; Table S2) was used in establishment of the chromatogrphy-mass spectrometry approach. Another QC sample (QC2) by pooling the equal volume of all test solutions was prepared for monitoring the system stability in multi-batch samples analysis for markers discovery.

### 3.3. Chromatographic Separation and MS Conditions

Holistic metabolites profiling was performed on an ACQUITY UPLC I-Class/Vion IMS-QTOF system (Waters, Milford, MA, USA). A BEH Shield RP18 column (2.1 × 100 mm, 1.7 μm) hyphenated with a VanGuard Pre-column (Waters; 2.1 × 50 mm, 1.7 μm) maintained at 35 °C was used for chromatographic separation. A binary mobile phase consisting of 0.1% FA in CH_3_CN (organic phase: A) and 0.1% FA in H_2_O (water phase: B) was employed at a flow rate of 0.3 mL/min following an optimized gradient program: 0–5 min, 15–23% (A); 5–15 min, 23–28% (A); 15–22 min, 28–29% (A); 22–33 min, 29–36% (A); 33–33.5 min, 36–55% (A); 33.5–37 min, 55–95% (A); and 37–40 min, 95% (A). A 3-min re-equilibration time was set between successive injections. Each 3 μL of the test solution was injected onto the column for UHPLC separation. A “purge–wash–purge” cycle was set on the autosampler, with 10% CH_3_CN-H_2_O (*v*/*v*) as the purge solvent and 50% CH_3_CN-H_2_O as the wash solvent, to minimize the carry-over between injections. The samples of PGF, PQF, and PNF, were injected randomly. The QC2 sample was injected after every eight samples to monitor the system stability.

The metabolomics data were acquired by a Vion IMS-QTOF mass spectrometer in the negative HDMS^E^ mode (Waters Corporation). The LockSpray ion source was equipped under the following parameters: capillary voltage, −1.0 kV; cone voltage, 20 V; source offset, 80 V; source temperature, 120 °C; desolvation gas temperature, 500 °C; desolvation gas flow (N_2_), 800 L/h; and cone gas flow (N_2_), 50 L/h. For the traveling wave IMS separation, default parameters were defined. The HDMS^E^ data covered a mass range of *m/z* 350–1500 at 0.3 s per scan. The low collision energy was set at 6 eV and the high-energy ramp was 80–120 eV. MS data calibration was conducted by constantly infusing the leucine enkephalin (LE; Sigma-Aldrich, St. Louis, MO, USA; 200 ng/mL) at a flow rate of 10 µL/min. Calibration of CCS was conducted according to the manufacture’s guideline using a mixture of calibrants [42]. Data acquisition was controlled by the UNIFI 1.9.3.0 software (Waters Corporation).

To verify the MS^2^ data of untargeted metabolomics-induced potential markers, targeted MS/MS experiments were performed on the Vion IMS-QTOF instrument after UHPLC separation. Dynamic retention windows were set based on their elution in the HDMS^E^ data of QC2. The ramp collision energy of 20–60 eV and 60–100 eV was set for the differential ions with *m/z* < 1000 and *m/z* > 1000, respectively, aiming to acquire rich product ions to support the structural elucidation.

### 3.4. Date Processing

The uncorrected HDMS^E^ data in Continuum of the samples (42 batches) and QC were initially corrected using UNIFI 1.9.3.0 by reference to *m/z* 554.2620. The corrected HDMS^E^ data were further processed by Progenesis QI 2.1 software (Waters Corporation). Isotope and adduct fusion were applied to reduce the number of detected metabolic features. The adduct ions, including [M − H]^−^, [M + FA − H]^−^, [M + Cl]^−^, [M + CH_3_COOH − H]^−^, [M − 2H]^2−^, [M − 2H + FA]^2−^, and [M − 2H + 2FA]^2−^, in the negative mode, were selected or self-edited. Efficient menu-guided processing (peak alignment and peak picking) could generate a data matrix, which involved the information of t_R_, *m/z*, normalized peak area, and CCS. The components, after “80% rule” and “30% variation” filtering, were used as the variables for multivariate statistical analysis using SIMCA-P 14.1 (Umetrics, Umea, Sweden) by PCA and OPLS-DA, with the data *Pareto* scaled. Those variables showing VIP > 3.0 were selected as the potential markers diagnostic for differentiating among PGF, PQF, and PNF, in the current work.

## 4. Conclusions

Aiming to elucidate the metabolome difference among the flower buds of three congeneric *Panax* species (PGF, PQF, and PNF), a UHPLC/IM-QTOF-MS-based untargeted metabolomics approach was established. Well resolution of ginsenosides was achievable on a BEH Shield RP18 column within 37 min using 0.1% FA in acetonitrile and 0.1% FA in water as the mobile phase. Sensitive and stable monitoring of ginsenosides was performed in the negative HDMS^E^ mode on the Vion IMS-QTOF high-resolution mass spectrometer (capillary voltage: 1.0 kV; cone voltage: 20 V; ramp collision energy: 80–120 eV). The developed UHPLC/IM-QTOF-HDMS^E^ approach enabled three-dimensional separations (RP, IMS, and MS) offering four-dimensional information (t_R_, CCS, MS^1^, and MS^2^), thus ensuring in-depth profiling and characterization of herbal multi-components. Using the standardized metabolomics workflows, after analyzing 42 batches of samples, 42 metabolic features corresponding to 32 potential marker compounds (involving 17 characteristic components and 15 differential compounds) were discovered. By multiple MS data interpretation techniques, six major markers were identified as Rb3, Ra1, isomer of m-Rc/m-Rb2/m-Rb3, isomer of Ra1/Ra2, Rb1, and isomer of Ra3. Additionally, three markers (Ra1, isomer of Ra1/Ra2, and isomer of Ra3), could be characteristic for PNF. To our knowledge, it is the first report that systematically compares the metabolome difference among PGF, PQF, and PNF. The established approach can be extensively applied to the quality control of TCM that contain similar metabolomes.

## Figures and Tables

**Table 1:** Information of 32 ginsenoside markers for differentiating among PGF, PQF, and PNF. L: low level; M: medium level; H: high level.

No.	VIP	t_R_ (min)	*m/z*	CCS (Å^2^)	Identification	PGF	PQF	PNF
M1	8.59	24.76	1077.5881	357.30	ginsenoside Rb3	L	H	M
M2 *	7.84	22.09	1209.6274	355.12	ginsenoside Ra1	L	L	H
M3	6.97	27.74	1163.5880	370.72	isomer of m-Rc/m-Rb2/m-Rb3	L	M	H
M4 *	6.63	25.37	1209.6274	356.45	isomer of ginsenoside Ra1/Ra2	L	L	H
M5	6.53	19.71	1107.5973	350.29	ginsenoside Rb1	M	L	H
M6 *	6.43	17.52	1239.6379	283.47	isomer of ginsenoside Ra3	L	L	H
M7 *	5.25	19.42	1209.6274	358.21	isomer of ginsenoside Ra1/Ra2	L	L	H
M8 *	5.21	21.07	1209.6274	351.82	isomer of ginsenoside Ra1/Ra2	L	L	H
M9 *	5.06	20.97	1325.6383	368.56	isomer of m-Ra3	L	L	H
M10 *	4.61	22.80	1341.6696	379.05	notoginsenoside Q/S or isomer	L	L	H
M11	4.46	29.77	1249.5896	358.00	dimal-Rc/Rb2/Rb3 or isomer	L	H	M
M12	4.41	27.06	1163.5885	377.51	isomer of m-Rc/m-Rb2/m-Rb3	L	H	M
M13 *	4.33	16.82	1341.6696	372.35	notoginsenoside Q/S or isomer	L	L	H
M14	4.00	25.70	1163.5880	347.14	isomer of m-Rc/m-Rb2/m-Rb3	L	M	H
M15	3.85	25.83	1193.5976	357.85	isomer of m-Rb1	H	M	L
M16	3.84	25.12	1163.5878	337.60	isomer of m-Rc/m-Rb2/m-Rb3	M	M	H
M17 *	3.82	26.37	1295.6327	365.60	m-Ra2 or isomer	L	L	H
M18	3.79	30.19	1249.5886	365.80	dimal-Rc/Rb2/Rb3 or isomer	L	H	M
M19	3.78	26.75	1163.5880	337.60	m-Rb3	L	H	M
M20 *	3.76	19.54	1341.6696	375.59	notoginsenoside Q/S or isomer	L	L	H
M21	3.72	5.35	695.1473	229.84	m-kaempferol-GlcGlc	M	H	L
M22	3.51	29.51	1163.5880	371.94	isomer of m-Rc/m-Rb2/m-Rb3	M	H	L
M23	3.50	21.91	1077.5879	338.39	ginsenoside Rc	L	M	H
M24 *	3.37	17.89	815.4830	301.49	ginsenoside F3	H	L	L
M25 *	3.36	18.71	1209.6274	351.14	isomer of ginsenoside Ra1/Ra2	L	L	H
M26	3.33	23.90	1077.5879	360.22	ginsenoside Rb2	M	H	M
M27 *	3.29	29.13	793.4395	282.26	chikusetsusaponin IVa	M	H	L
M28 *	3.21	19.55	716.3369	354.60	unknown	L	L	H
M29 *	3.20	13.10	845.4921	298.26	24(*R*)-pseudoginsenoside F11	L	H	L
M30 *	3.11	14.72	1371.6802	386.06	notoginsenoside D/T or isomer	L	L	H
M31	3.09	15.00	845.4921	302.65	ginsenoside Rf	H	L	M
M32 *	3.03	23.72	1295.6327	362.76	m-Ra2 or isomer	L	L	H

*: Considered as the characteristic components.

## Supplementary Materials

The following are available online: Figure S1 Comparison of the influence of formic acid (0.1% FA; A) and ammonium acetate (3 mM AA; B) as the additive in mobile phase for the resolution of ginsenosides from a QC sample. Optimal gradient elution programs were used for each determination. It clearly shows more peaks could be resolved by adding 0.1% FA in the water phase. Figure S2 Comparison of the influence of temperature (25–40 °C) on the BEH Shield RP18 column for the resolution of ginsenosides from a QC1 sample. Figure S3 Comparison of different levels of ramp collision energies in the negative mode for the CID-MS^2^ fragmentation of ginsenosides using Rb1 and Re as the representatives. Figure S4 Comparison of the base peak chromatograms of QC1 sample obtained by MS^E^ and HDMS^E^ in the negative mode. Figure S5 PCA score plot by analysis of 42 batches of samples. Table S1 Information of 39 ginsenoside reference compounds used in this work. Table S2 Information of 42 batches of the flower bud samples of *P. ginseng* (PGF), *P. quinquefolius* (PQF), and *P. notoginseng* (PNF). Table S3 Assignment of 42 ions with VIP > 3.0 to 32 marker compounds.
