# Supplementary material for: Simultaneous Profiling and Holistic Comparison of the Metabolomes among the Flower Buds of Panax ginseng, Panax quinquefolius, and Panax notoginseng by UHPLC/IM-QTOF-HDMSE-Based Metabolomics Analysis
Source: Molecules. 2019 Jun 11;24(11):2188. doi: 10.3390/molecules24112188 (PMC6600391; doi:10.3390/molecules24112188)
Supplement: Supplementary file 1 [file molecules-24-02188-s001.pdf]

<**Molecules**>

SUPPLEMENTARY MATERIALS

Simultaneous profiling and holistic comparison of the metabolomes among the flower buds of *Panax ginseng*, *Panax quinquefolius*, and *Panax notoginseng* by UHPLC/IM-QTOF-HDMS^{E}-based metabolomics analysis

Li Jia [1,2,†], Tiantian Zuo [1,2,†], Chunxia Zhang [1,2,†], Weiwei Li [1,2], Hongda Wang [1,2], Ying Hu [1,2], Xiaoyan Wang [1,2], Yuexin Qian [1,2], Wenzhi Yang [1,2,*] and Heshui Yu [1,3,*]

[1] Tianjin State Key Laboratory of Modern Chinese Medicine, Tianjin University of Traditional Chinese Medicine, 312 Anshanxi Road, Tianjin 300193, China;

[2] Tianjin Key Laboratory of TCM Chemistry and Analysis, Tianjin University of Traditional Chinese Medicine, 312 Anshanxi Road, Tianjin 300193, China

[3] College of Pharmaceutical Engineering of Traditional Chinese Medicine, Tianjin University of Traditional Chinese Medicine, Tianjin 300193, China

[*] Correspondence: wzyang0504@tjutcm.edu.cn, Tel.: +86-022-5979-1833 (W.Y.); hs_yu08@163.com (H.Y.)

[†] These authors contributed equally to this work.

Contents

Figure S1 Comparison of the influence of formic acid (0.1% FA; **A**) and ammonium acetate (3 mM AA; **B**) as the additive in mobile phase for the resolution of ginsenosides from a QC sample. Optimal gradient elution programs were used for each determination. It clearly shows more peaks could be resolved by adding 0.1%FA in the water phase.

Figure S2 Comparison of the influence of temperature (25–40°C) on the BEH Shield RP18 column for the resolution of ginsenosides from a QC1 sample.

Figure S3 Comparison of different levels of ramp collision energies in the negative mode for the CID-MS^2 fragmentation of ginsenosides using Rb1 and Re as the representatives.

Figure S4 Comparison of the base peak chromatograms of QC1 sample obtained by MS^E and $HDMS^E$ in the negative mode.

Figure S5 PCA score plot by analysis of 42 batches of samples.

Table S1 Information of 39 ginsenoside reference compounds used in this work.

Table S2 Information of 42 batches of the flower bud samples of *P. ginseng* (PGF), *P. quinquefolius* (PQF), and *P. notoginseng* (PNF).

Table S3 Assignment of 42 ions with VIP > 3.0 to 32 marker compounds.

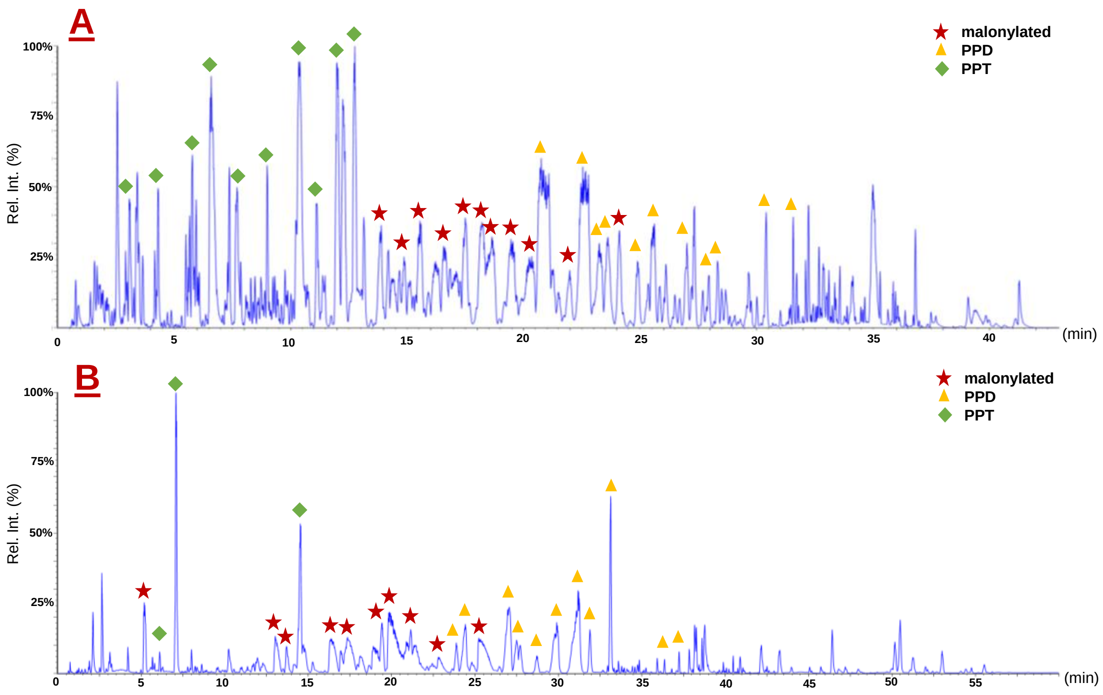

Figure S1 Comparison of the influence of formic acid (0.1% FA; **A**) and ammonium acetate (3 mM AA; **B**) as the additive in mobile phase for the resolution of ginsenosides from a QC sample. Optimal gradient elution programs were used for each determination. It clearly shows more peaks could be resolved by adding 0.1%FA in the water phase.

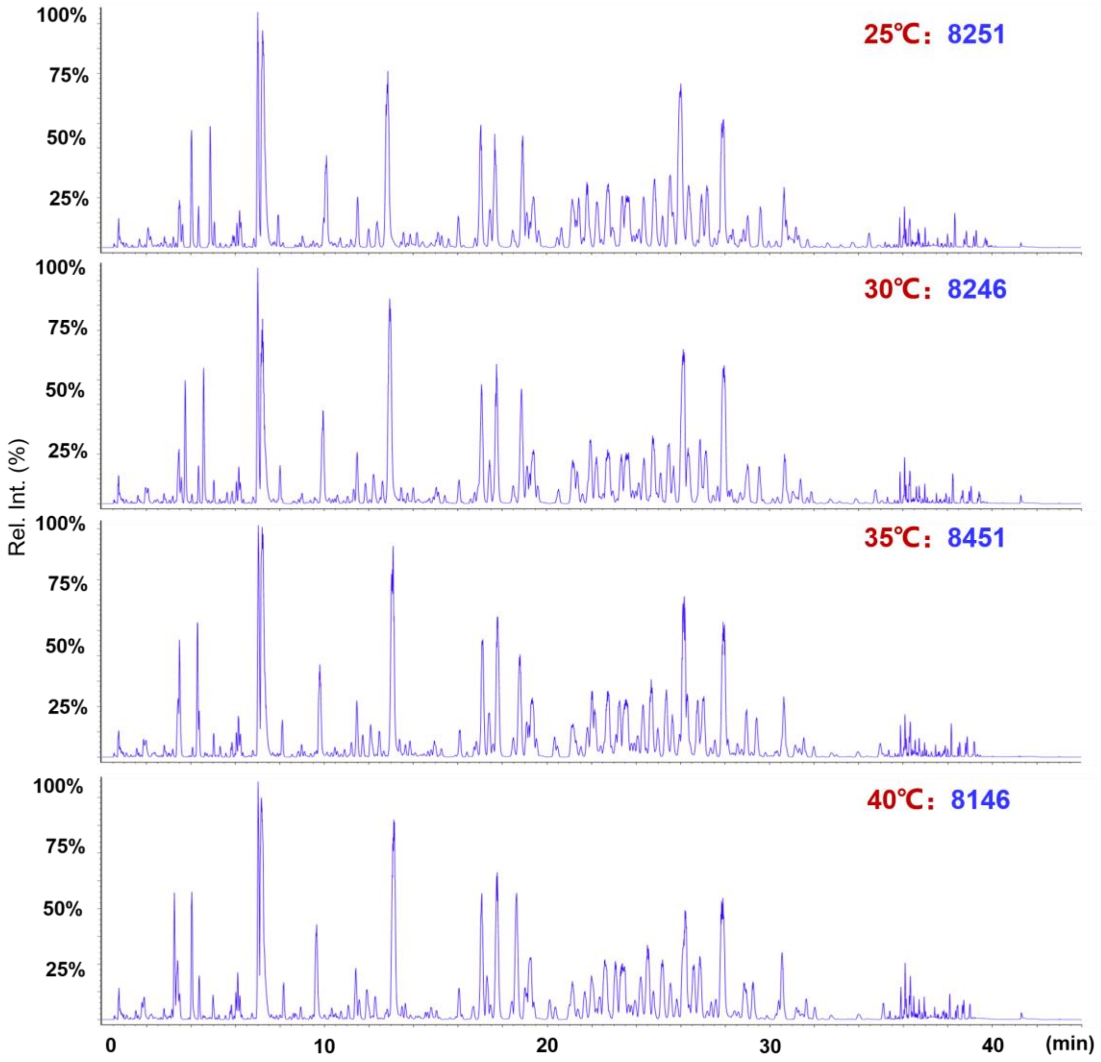

Figure S2 Comparison of the influence of temperature (25–40°C) on the BEH Shield RP18 column for the resolution of ginsenosides from a QC1 sample.

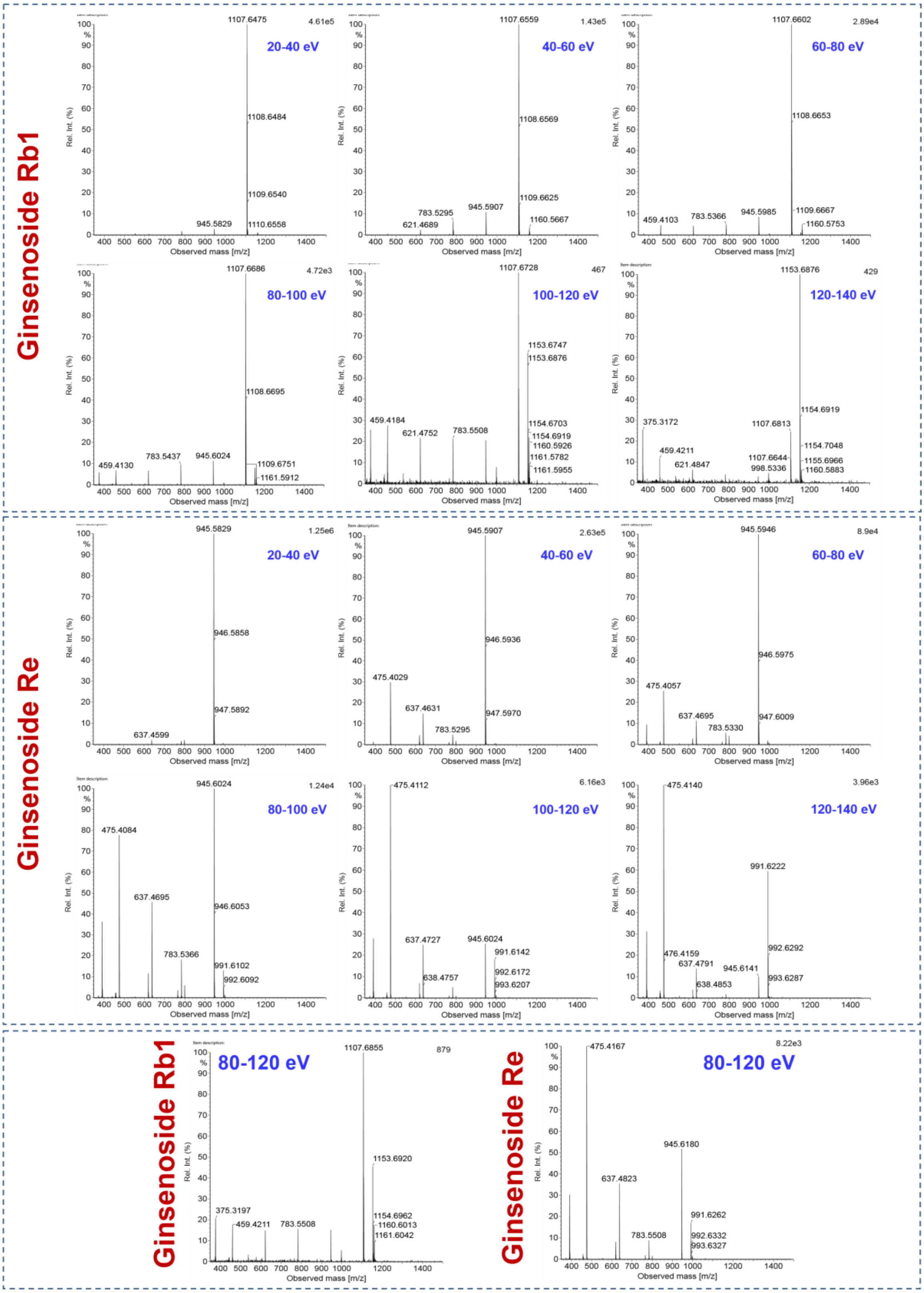

Figure S3 Comparison of different levels of ramp collision energies in the negative mode for the CID-MS^2 fragmentation of ginsenosides using Rb1 and Re as the representatives.

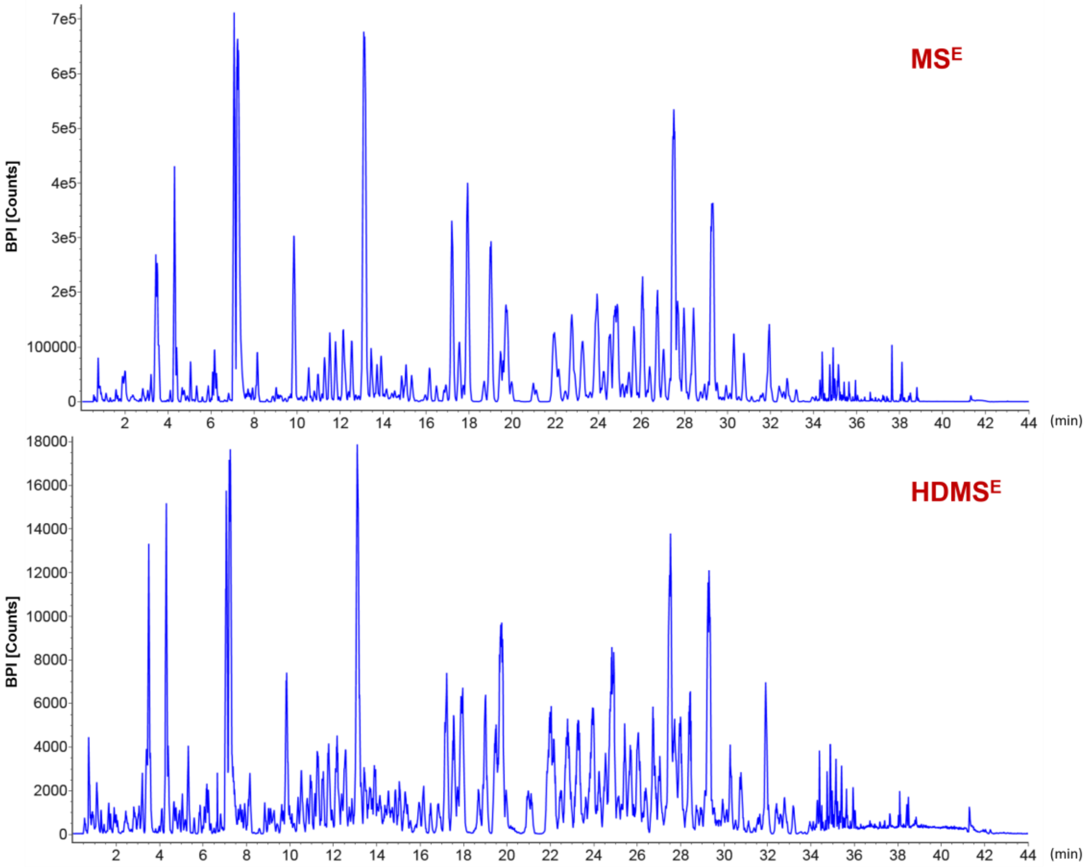

Figure S4 Comparison of the base peak chromatograms of QC1 sample obtained by MS^E and $HDMS^E$ in the negative mode.

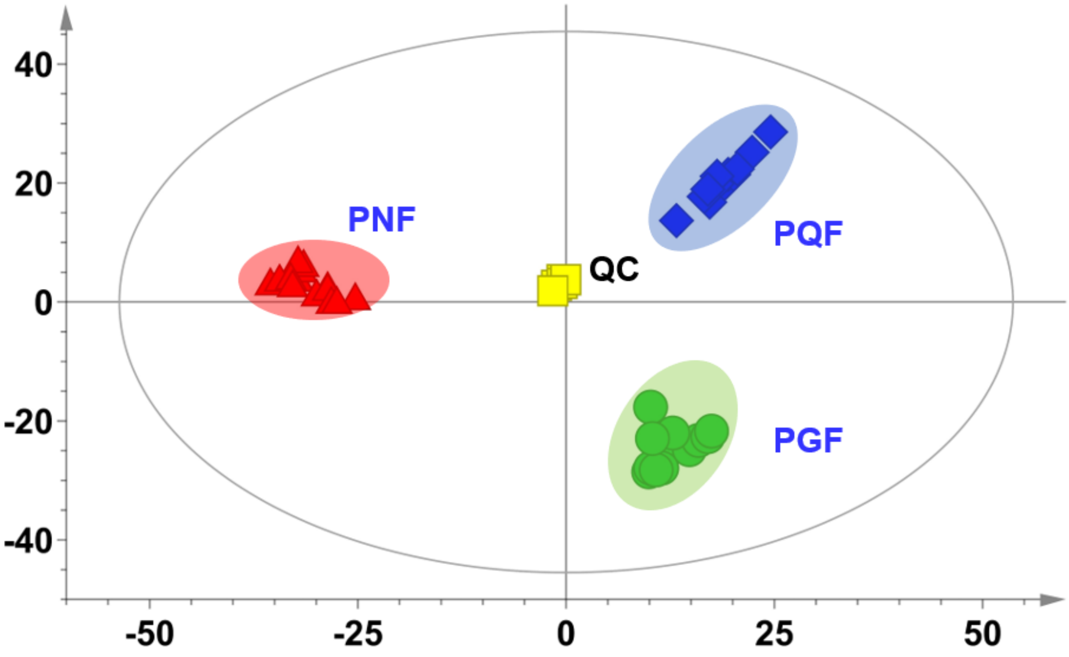

Figure S5 PCA score plot by analysis of 42 batches of samples.

Table S1 Information of 39 ginsenoside reference compounds used in this work.

No.	Compound	Formula	Exact Mass
**1**	vinaginsenoside R4	$C_{48}H_{82}O_{19}$	962.5450
**2**	ginsenoside Re	$C_{48}H_{82}O_{18}$	946.5501
**3**	ginsenoside Rf	$C_{42}H_{72}O_{14}$	800.4922
**4**	ginsenoside Rg1	$C_{42}H_{72}O_{14}$	800.4922
**5**	ginsenoside Rg2	$C_{42}H_{72}O_{13}$	784.4973
**6**	ginsenoside Rh1	$C_{36}H_{62}O_{9}$	638.4394
**7**	20(*R*)-ginsenoside Rh1	$C_{36}H_{62}O_{9}$	638.4394
**8**	notoginsenoside R1	$C_{47}H_{80}O_{18}$	932.5345
**9**	notoginsenoside R2	$C_{41}H_{70}O_{13}$	770.4816
**10**	ginsenoside F1	$C_{36}H_{62}O_{9}$	638.4394
**11**	ginsenoside F3	$C_{41}H_{70}O_{13}$	770.4816
**12**	20(*S*)-sanchirhinoside A3	$C_{41}H_{70}O_{13}$	770.4816
**13**	20(*R*)-notoginsenoside R2	$C_{41}H_{70}O_{13}$	770.4816
**14**	malonylfloralginsenoside Re1	$C_{48}H_{82}O_{19}$	1032.5505
**15**	ginsenoside Rb1	$C_{54}H_{92}O_{23}$	1108.6029
**16**	ginsenoside Rb2	$C_{53}H_{90}O_{22}$	1078.5924
**17**	ginsenoside Rc	$C_{53}H_{90}O_{22}$	1078.5924
**18**	ginsenoside Rd	$C_{48}H_{82}O_{18}$	946.5501
**19**	malonylginsenoside Rb1	$C_{57}H_{94}O_{26}$	1194.6033
**20**	malonylginsenoside Rb2	$C_{56}H_{92}O_{25}$	1164.5928
**21**	malonylginsenoside Rc	$C_{56}H_{92}O_{25}$	1164.5928
**22**	malonylginsenoside Rd	$C_{51}H_{84}O_{21}$	1032.5505
**23**	20(*R*)-ginsenoside Rg3	$C_{42}H_{72}O_{13}$	784.4973
**24**	ginsenoside Rb3	$C_{53}H_{90}O_{22}$	1078.5924
**25**	ginsenoside F2	$C_{42}H_{72}O_{13}$	784.4973
**26**	notoginsenoside K	$C_{48}H_{82}O_{18}$	946.5501
**27**	notoginsenoside R4	$C_{59}H_{100}O_{27}$	1240.6452
**28**	notoginsenoside T	$C_{64}H_{108}O_{31}$	1372.6875
**29**	ginsenoside Ra1	$C_{58}H_{98}O_{26}$	1210.6346
**30**	ginsenoside Ra2	$C_{58}H_{98}O_{26}$	1210.6346
**31**	20(*S*)-ginsenoside Rg3	$C_{42}H_{72}O_{13}$	784.4973
**32**	20(*S*)-ginsenoside Rh2	$C_{36}H_{62}O_{8}$	622.4445
**33**	ginsenoside Ro	$C_{48}H_{76}O_{19}$	956.4981
**34**	chikusetsusaponin IVa	$C_{42}H_{66}O_{14}$	794.4453
**35**	24(*R*)-pseudoginsenoside F11	$C_{42}H_{72}O_{14}$	800.4922
**34**	24(*R*)-pseudoginsenoside Rt5	$C_{36}H_{62}O_{10}$	654.4343
**37**	ginsenoside Rk1	$C_{42}H_{70}O_{12}$	766.4867
**38**	ginsenoside Rg5	$C_{42}H_{70}O_{12}$	766.4867
**39**	5,6-didehydroginsenoside Rb1	$C_{54}H_{90}O_{23}$	1106.5873

Table S2 Information of 45 batches of the flower bud samples of *P. ginseng* (PGF), *P. quinquefolius* (PQF), and *P. notoginseng* (PNF).

Species	No	Producing origin	Harvesting time
**PGF**	PGF-1	Mudanjiang, Heilongjiang	2018.09
	PGF-2	Mudanjiang, Heilongjiang	2018.09
	PGF-3	Changbaishan, Jilin	2018.08
	PGF-4	Baishan, Jiling	2018.09
	PGF-5	Jingyu,Baishan, Jilin	2018.09
	PGF-6	Jingyu,Baishan, Jilin	2018.09
	PGF-7	Jingyu,Baishan, Jilin	2018.09
	PGF-8	Jingyu,Baishan, Jilin	2018.09
	PGF-9	Jingyu,Baishan, Jilin	2018.09
	PGF-10	Changbaishan, Jilin	2018.09
	PGF-11	Baishan, Jilin	2018.09
	PGF-12	Changbaishan, Jilin	2018.09
	PGF-13	Changbaishan, Jilin	2018.09
	PGF-14	Changbaishan,Jilin	2018.09
**PQF**	PQF-1	Mudanjiang, Heilongjiang	2018.09
	PQF-2	Changbaishan, Jilin	2018.09
	PQF-3	Changbaishan, Jilin	2018.09
	PQF-4	Weihai, Shandong	2018.09
	PQF-5	Tonghua, Jilin	2018.09
	PQF-6	Changbaishan, Jilin	2018.09
	PQF-7	Changbaishan, Jilin	2018.09
	PQF-8	Changbaishan, Jilin	2018.09
	PQF-9	Changbaishan, Jilin	2018.09
	PQF-10	Baishan, Jilin	2018.09
	PQF-11	Changbaishan, Jilin	2018.09
	PQF-12	Changbaishan, Jilin	2018.09
	PQF-13	Changbaishan, Jilin	2018.09
	PQF-14	Jingyu,Baishan, Jilin	2018.09
**PNF**	PNF-1	Wenshan, Yunnan	2018.04
	PNF-2	Wenshan, Yunnan	2018.04
	PNF-3	Wenshan, Yunnan	2018.04
	PNF-4	Wenshan, Yunnan	2018.04
	PNF-5	Wenshan, Yunnan	2018.09
	PNF-6	Wenshan, Yunnan	2018.09
	PNF-7	Wenshan, Yunnan	2018.09
	PNF-8	Wenshan, Yunnan	2018.09
	PNF-9	Wenshan, Yunnan	2018.09
	PNF-10	Wenshan, Yunnan	2018.09
	PNF-11	Wenshan, Yunnan	2018.09

PNF-12	Wenshan, Yunnan	2018.09
PNF-13	Wenshan, Yunnan	2018.09
PNF-14	Shilin, Yunnan	2018.09

Table S3 Assignment of 42 ions with VIP > 3.0 to 32 marker compounds.

No.	t_R (min)	m/z	CCS (Å^2)	VIP	Matched differential ions (*m/z*/t_R/CCS)	MS^2 fragments	Identification	PGF	PQF	PNF
**1**	24.76	1077.5881	357.30	8.59	**1124.5970** (24.95/ 358.67): isotope peak of *m/z* 1123.5936<br>**1124.5973** (24.92/ 358.67): isotope peak of *m/z* 1123.5936<br>**1078.0888** (24.88 / 257.89): isotope peak of *m/z* 1077.5881<br>**1078.0888** (24.81 / 257.89): isotope peak of *m/z* 1077.5881<br>**561.2917** (24.79 /205.51): [M–2H+HCOOH]$^{2-}$	945.5434, 783.4883, 765.4808, 621.4354, 459.3828, 375.2885	ginsenoside Rb3	L	H	M
**2***	22.09	1209.6274	355.12	7.84	**1209.6312** (22.08 / 355.14): [M–H]$^-$<br>**1143.6086** (22.02 / 531.44): unknown<br>**627.3131** (22.13 / 437.73): [M–2H+HCOOH]$^{2-}$	1209.6245, 1149.6031, 1077.5842, 945.5381, 915.5353, 783.4870, 765.4803, 621.4370, 459.3838, 375.2913	ginsenoside Ra1	L	L	H
**3**	27.74	1163.5880	370.72	6.97	**1165.6016** (27.79 / 371.77): isotope peak of *m/z* 1163.5880	1077.5848, 945.5417, 783.4892, 621.4369, 459.3843	isomer of m-Rc/m-Rb2/m-Rb3	L	M	H
**4***	25.37	1209.6274	356.45	6.63	**604.3103** (25.39 / 428.72): [M–2H]$^{2-}$	1163.5925, 1119.5939, 1077.5860, 1059.5678, 621.4327, 459.3890	isomer of ginsenoside Ra1/Ra2	L	L	H

**5**	19.71	1107.5973	350.29	6.53	**576.2970** (19.70 / 212.42): $[M–2H+HCOOH]^{2–}$	1107.5936, 945.5459, 783.4922, 621.4382, 459.3844	ginsenoside Rb1	M	L	H
**6***	17.52	1239.6379	283.47	6.43	**1239.6418** (17.52 / 362.95): $[M–H]^{–}$<br>**642.3183** (17.54 / 442.18): $[M–2H+HCOOH]^{2–}$	1107.5995, 945.5605, 783.4899, 621.4371, 459.3843	isomer of ginsenoside Ra3	L	L	H
**7***	19.42	1209.6274	358.21	5.25	**627.3131** (19.43 / 432.96): $[M–2H+HCOOH]^{2–}$	1209.6320, 1107.5897, 1077.5865, 945.5334, 783.5136, 621.4390	isomer of ginsenoside Ra1/Ra2	L	L	H
**8***	21.07	1209.6274	351.82	5.21	**1209.6312** (21.13 / 363.22): $[M–H]^{–}$	1209.6338, 1077.5842, 945.5445, 915.5314, 783.4913, 765.4796, 621.4348, 459.3869, 375.2889	isomer of ginsenoside Ra1/Ra2	L	L	H
**9***	20.97	1325.6383	368.56	5.06	**663.3236** (21.01 / 451.33): $[M:]^{2–}$	1281.6472, 1239.6372, 1221.6271, 1107.5953, 1089.5846, 1041.5614, 945.5422, 927.5338, 783.4901, 765.4790, 621.4374, 603.4276, 459.3849, 375.2900	isomer of m-Ra3	L	L	H
**10***	22.80	1341.6696	379.05	4.61	**693.3343** (22.88 / 445.92): $[M–2H+HCOOH]^{2–}$	1209.6208, 1077.5893, 783.4950	notoginsenoside Q/S or isomer	L	L	H
**11**	29.77	1249.5896	358.00	4.46	**603.3023** (29.86 / 419.27): $[M:]^{2–}$	1249.5896, 1205.5993, 1077.5853, 1059.5718, 945.5423, 915.5315, 825.5047, 783.4920, 765.4840, 621.4360, 603.4242, 537.3341, 459.3845,	dimal-Rc/Rb2/Rb3 or isomer	L	H	M

						375.2916				
**12**	27.06	1163.5885	377.51	4.41	**1165.6016** (27.17 / 371.77): isotope peak of *m/z* 1163.5885	1119.5855, 1079.5896, 1077.5825, 945.5395, 783.4894, 765.4806, 621.4350, 459.3839	isomer of m-Rc/m-Rb2/m-Rb3	L	H	M
**13***	16.82	1341.6696	372.35	4.33	**693.3344** (16.84 / 450.71): $[M{-}2H{+}HCOOH]^{2-}$	1341.6617, 1209.6290, 1077.5856, 945.5393, 783.4925, 765.4822, 621.4396, 459.3801	notoginsenoside Q/S or isomer	L	L	H
**14**	25.70	1163.5880	347.14	4.00	**582.2970** (25.88 / 207.55): $[M{:}]^{2-}$	1119.5952, 1077.5839, 945.5417, 783.4890, 621.4367, 459.3845	isomer of m-Rc/m-Rb2/m-Rb3	L	M	H
**15**	25.83	1193.5976	357.85	3.85	**597.3024** (25.91 / 428.88): $[M{:}]^{2-}$	1193.5976, 1163.5869, 1119.6014, 597.3024, 582.2961, 559.2945	isomer of m-Rb1	H	M	L
**16**	25.12	1163.5878	337.60	3.84	**582.2970** (25.19 / 209.93): $[M{:}]^{2-}$	1077.5839, 1059.5745, 945.5441, 915.5295, 783.4885, 765.4781, 621.4337, 459.3850	isomer of m-Rc/m-Rb2/m-Rb3	M	M	H
**17***	26.37	1295.6327	365.60	3.82	**648.3181** (26.45 / 437.28): $[M{:}]^{2-}$	1251.6390, 1209.6268, 1191.6165, 1077.5838, 1059.5746, 945.5430, 915.5335, 783.4892, 765.4789, 621.4363, 603.4274, 459.3846, 375.2891	m-Ra2 or isomer	L	L	H

**18**	30.19	1249.5886	365.80	3.79	**1165.6036** (30.27 / 369.94): isotope peak of *m/z* 1163.5885 [M–H–Mal.]$^{–}$	1119.5993, 1077.5852, 945.5409, 783.4903, 621.4358, 603.4263, 459.3850	dimal-Rc/Rb2/Rb3 or isomer	L	H	M
**19**	26.75	1163.5880	337.60	3.78	**582.2970** (26.85/ 410.32): [M:]$^{2–}$ **1121.6058** (26.79 / 355.97): isotope peak of *m/z* 1119.5972 [M–H–CO_2]$^{–}$	1077.5845, 1059.5744, 945.5424, 783.4895, 375.2889	m-Rb3	L	H	M
**20***	19.54	1341.6696	375.59	3.76	**1342.6777** (19.58 / 378.34): isotope peak of *m/z* 1341.6696 **1255.6363** (19.47 / 362.81): isotope peak of *m/z* 1341.6696 [M–H–Mal.]$^{–}$ **693.3343** (19.60 / 460.34): [M–2H+HCOOH]$^{2–}$	1341.6664, 1209.6270, 1077.5838, 1047.5723, 945.5428, 915.5301, 783.4883, 765.4779, 621.4407, 603.4359, 459.3846, 375.2945	notoginsenoside Q/S or isomer	L	L	H
**21**	5.35	695.1473	229.84	3.72	**651.1575** (5.36 / 237.92): [M–H-CO_2] $^{–}$	651.1567, 609.1474, 429.0839, 327.0512, 285.0404/284.0326, 255.0299, 227.0350, 211.0398, 151.0038	m-kaempferol-GlcGlc	M	H	L
**22**	29.51	1163.5880	371.94	3.51	**582.2971** (29.57 / 415.02): [M:]$^{2–}$	1119.5968, 1077.5829, 1059.5739, 945.5470, 915.5283, 783.4916, 765.4834, 621.4382, 459.3857, 375.2916	isomer of m-Rc/m-Rb2/m-Rb3	M	H	L
**23**	21.91	1077.5879	338.39	3.50	**561.2917** (21.98 / 406.11): [M–2H+HCOOH]$^{2–}$	1149.6087, 1077.5847, 945.5420, 783.4865, 621.4409, 537.3420, 459.3848, 375.2912	ginsenoside Rc	L	M	H

**24***	17.89	815.4830	301.49	3.37	**769.4765** (17.92 / 301.91): [M–H] ⁻	815.4783, 769.4735, 637.4317, 475.3792	ginsenoside F3	H	L	L
**25***	18.71	1209.6274	351.14	3.36	**627.3130** (18.74 / 428.21): $[M–2H+HCOOH]^{2–}$	1209.6313, 1077.5825, 945.5443, 783.4906, 621.4291, 459.3856, 375.2872	isomer of ginsenoside Ra1/Ra2	L	L	H
**26**	23.90	1077.5879	360.22	3.33	**561.2916** (23.92 / 203.14): $[M–2H+HCOOH]^{2–}$	1077.5838, 945.5543, 783.4890, 621.4353, 459.3855	ginsenoside Rb2	M	H	M
**27***	29.13	793.4395	282.26	3.29	**793.4395** (29.20 / 283.56): [M–H] ⁻	793.4370, 775.9442, 733.4243, 673.3955, 631.3846, 613.3744, 569.3849, 497.3650, 455.3537, 437.3432	chikusetsusaponin IVa	M	H	L
**28***	19.55	716.3369	354.60	3.21	**716.3369** (19.58 / 464.72): $[M–H]^{–}$	671.3352, 621.4366, 603.4279, 539.2919, 537.3458, 459.3848, 457.2608, 375.2094	unknown	L	L	H
**29***	13.10	845.4921	298.26	3.20	**801.4927** (13.09 / 304.07): isotope peak of *m/z* 799.4836 [M–H] ⁻	799.4836, 653.4272, 637.4288, 491.3761, 415.3231	24(*R*)-pseudoginsenoside F11	L	H	L
**30***	14.72	1371.6802	386.06	3.11	**708.3396** (14.73 / 455.22): $[M–2H+HCOOH]^{2–}$	1371.6858, 1329.6417, 1107.5983, 945.5428, 783.4909, 459.3831, 353.1094	notoginsenoside D/T or isomer	L	L	H
**31**	15.00	845.4921	302.65	3.09	**845.4921** (14.98 / 300.91): $[M–H+HCOOH]^{–}$	845.4882, 799.4834, 781.8612, 637.4314, 475.3787	ginsenoside Rf	H	L	M

**32***	23.72	1295.6327	362.76	3.03	**648.3182** (23.72 / 442.06): $[M:]^{2-}$	1251.6577, 1209.6277, 1191.6168, 1077.5864, 1059.5753, 1047.5689, 945.5400, 915.5307, 783.4911, 765.4799, 621.4360, 603.4288, 459.3847, 375.2903	m-Ra2 or isomer	L	L	H

***:** characteristic components.
